# Lycorine ameliorates isoproterenol-induced cardiac dysfunction mainly via inhibiting inflammation, fibrosis, oxidative stress and apoptosis

**DOI:** 10.1080/21655979.2021.1967019

**Published:** 2021-09-13

**Authors:** Ji Wu, Yang Fu, Ying-xing Wu, Zu-xiang Wu, Zhen-hua Wang, Ping Li

**Affiliations:** Department of Cardiovascular Medicine, The Second Affiliated Hospital of Nanchang University, Nanchang, Jiangxi, China

**Keywords:** Heart failure, cardiac dysfunction, isoproterenol, lycorine, alkaloid

## Abstract

Alleviating cardiac dysfunction improves the prognosis of heart failure patients. Lycorine is an alkaloid with several beneficial biological properties. Here, we used mice to evaluate the effect of lycorine on cardiac dysfunction elicited by isoproterenol. Mice were divided into four groups: control, lycorine, isoproterenol, and isoproterenol + lycorine. Mice in the combined group were treated daily with 10 mg/kg isoproterenol intraperitoneally for 2 weeks and 5 mg/kg lycorine was given simultaneously intraperitoneally for 4 weeks. Cardiac structure and function were assessed by echocardiography, hematoxylin and eosin staining, and Masson’s trichrome staining. Isoproterenol-induced cardiac dysfunction and histopathological injury that was significantly improved by treatment with lycorine. Western blotting and the quantitative real-time polymerase chain reaction were used to explore the molecular mechanisms of these effects. Levels of the inflammatory cytokines, interleukin (IL)-1β, IL-6, and tumor necrosis factor-α, were increased by treatment with isoproterenol; these increases were significantly reduced by lycorine, with involvement of the NF-κB signaling pathway. The fibrotic factors, collagen I and collagen III, were increased by isoproterenol and decreased by treatment with lycorine through inhibiting activation of the Smad signaling pathway. In addition, lycorine alleviated oxidative stress as evidenced by a reduction in total reactive oxygen species in the isoproterenol + lycorine group compared to the isoproterenol group. Lycorine exerted an anti-apoptotic effect as evidenced by upregulating Bcl-2 and downregulating Bax. Overall, our findings demonstrate that lycorine protects against cardiac dysfunction induced by isoproterenol by inhibiting inflammation, fibrosis, oxidative stress, and apoptosis.

## Introduction

Heart failure (HF) is considered a serious and complex result of cardiovascular disease. It is characterized by impairment of cardiac systolic and diastolic functions, as well as irreversible cardiac dysfunction [1]. HF is a common end-phase of several cardiovascular diseases including hypertension, acute myocardial infarction, septic cardiomyopathy, and diabetic cardiomyopathy [2–6]. Alleviating cardiac dysfunction markedly prolongs the survival of patients with HF. Despite several effective treatments for HF, its morbidity and mortality remain at a high level, which contributes to substantial burdens to the family and society [7]. Thus, discovering new effective therapeutic approaches for HF is an urgent need.

Lycorine (LYC), one component of Amaryllidaceae, is an alkaloid that displays various beneficial functions in some disease models, including anti-inflammation, anti-fibrosis, antiviral, anti-aging, and anti-tumor effects [8–12]. Recently, numerous studies focused on investigating the antitumor feature of LYC in colorectal cancer, gastric cancer, bladder cancer, lung cancer, breast cancer, melanoma, multiple myeloma, osteosarcoma, hematological malignancies [13–21]. After administration of LYC, cancer cells died selectively in large numbers because of apoptosis [21]. Another study reported that LYC can inhibit the growth of flavivirus. The possible antiviral mechanism of LYC might derive from the suppression of viral RNA replication [22]. Similar antiviral effects of LYC were also discovered on Zika virus and the novel coronavirus [23,24]. The anti-aging property of LYC indicates that it may hold therapeutic potential for treating age-associated diseases [11]. Moreover, studies reported that LYC can attenuate lipopolysaccharide-induced acute lung injury and bleomycin-induced pulmonary fibrosis by suppressing inflammation. This implies that LYC might serve as a new potential therapeutic agent to alleviate inflammation and fibrosis in pulmonary diseases [25,26]. Due to these beneficial biological activities of LYC, we conducted a study to explore its effect on isoproterenol (ISO)-induced cardiac dysfunction. ISO, which acts as a nonselective beta adrenoceptor agonist, is well-known for inducing cardiac dysfunction and some related cardiovascular diseases [27].

Due to the facts that searching effective treatments for heart failure is urgent and lycorine displays benefical biological features like suppressing inflammation and fibrosis, so we wanted to investigate what effects of lycorine on HF. Therefore, we used ISO to induce cardiac dysfunction in mice and then treated mice with LYC in the present study. We also applied some experimental techniques to evaluate the effects and mechanism of LYC on ISO-induced cardiac dysfunction in mice.

## Materials and methods

### Chemicals and animals

A total of 36 male C57BL/6 J mice (6 weeks of age, weighing 20 ± 5 g), obtained from the Hunan SJA Laboratory Animal Company (Changsha, China), were used. LYC was purchased from Aladdin Biochemical Technology (Shanghai, China). ISO was purchased from Shanghai Harvest Pharmaceutical (Shanghai, China). All other reagents used in the present study were generic and generally commercially available.

### Animal model

**Figure 1.**
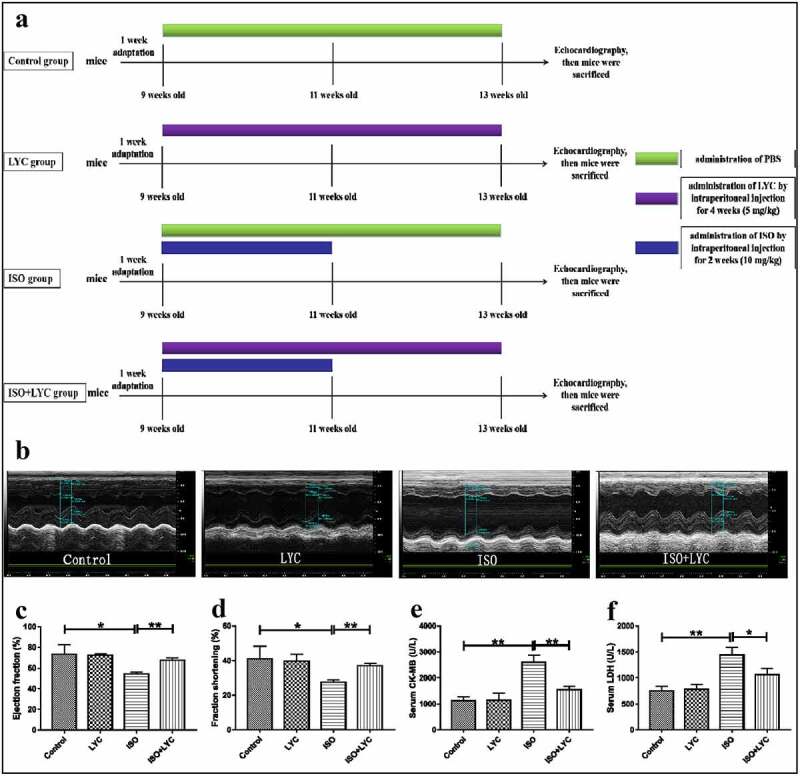
Treatment Of LYC Alleviated Cardiac Dysfunction Induced By ISO. (a) A Summary Of Animal Treatments Procedure. (b) Measurements Of Echocardiography In Different Groups Of Mice. (c,d) Ejection Fraction (%) And Fractional Shortening (%) Of Heart Were Evaluated By Echocardiography. E And F, The Levels Of Specific Cardiac Injury Biomarkers (CK-MB And LDH) In Plasma. *P < 0.05 And **P < 0.01 Mean Statistic Significance

Animals were housed with a 12-h light/dark cycle in the Animal Center of Jiangxi Medical of Nanchang University, with free access to food and water. The temperature of the environment was maintained at 24 ± 2°C with a relative humidity of 55 ± 5%. Mice were adapted to the feeding conditions for 1 week before any animal experimental operations. The mice were then randomized into four groups of eight as follows: (1) control: treated with phosphate-buffered saline (PBS) by intraperitoneal injection without any other treatments; (2) LYC: the LYC powder was dissolved in PBS and given to mice intraperitoneally daily for 4 weeks (5 mg/kg). (3) ISO: administered ISO daily for 2 weeks intraperitoneally (10 mg/kg); (4) ISO + LYC: in addition to receiving LYC for 4 weeks, these mice were given ISO simultaneously for 2 weeks (LYC, 5 mg/kg; ISO, 10 mg/kg). The way of our administering lycorine was in accordance with researches conducted by Qing Liang, Geng-Rui Xu and Katharina Schimmel[26,28,29]. An overview of the animal treatments is given in Figure 1(a).

### Echocardiography

Transthoracic echocardiography was performed to assess changes in cardiac function in each group using a Vevo770 (VisualSonics, Toronto, ON, Canada) equipped with a 30 Hz transducer. Mice were anesthetized via continuous inhalation of 2% isoflurane and fixed on a heating pad in a supine position. A short-axis view under M-mode tracings was selected to measure the internal dimensions of the left ventricle at both end-diastole and end-systole. Subsequently, the left ventricular ejection fraction and left ventricular fractional shortening were obtained by automatic calculations. Mean values of a minimum of three repeated measurements were applied to the final analysis.

### CK-MB and LDH activity assays

After echocardiography, the mice were sacrificed, heart tissue collected for further measurements, and whole blood was acquired from the right ventricle using vacuum tubes. Plasma was extracted from whole blood following centrifugation at 4000 g for 15 min at 4°C. Plasma levels of the cardiac injury markers, creatine kinase-MB (CK-MB) and lactate dehydrogenase (LDH), were detected by a Chemray240 automatic biochemical analyzer (Rayto Life Technology, Shenzhen, China).

### Detection of inflammatory cytokines (interleukin (IL)-1β, IL-6, and tumor necrosis factor (TNF-α)) and oxidative stress markers (malondialdehyde (MDA) and superoxide dismutase (SOD))

The levels of inflammatory cytokines in plasma were measured through specific mouse enzyme-linked immunosorbent assay kits for IL-1β (cat. no. 88–7013), IL-6 (cat. no. 88–7064), and TNF-α (cat. no. 88–7324) purchased from Invitrogen (Carlsbad, CA, USA). The MDA (cat. no. A003-1) and SOD (cat. no. A001-3) detection kits were acquired from Nanjing Jiancheng Bioengineering Institute (Nanjing, China).

### Histopathology

A 3% pentobarbital sodium was used to anaesthetize mice. Then, the heart tissue was removed and washed three times in ice-cold saline. One part of the left ventricle was fixed in 4% formaldehyde, then cut into 5-μm slices of different depths. The heart tissue slices were used to assess histopathological changes following hematoxylin and eosin, Masson’s trichrome, and dihydroethidium (DHE) staining. The remaining heart tissue was stored at −80 °C and used for studies exploring the molecular mechanisms of LYC.

### Quantitative real-time polymerase chain reaction (qRT-PCR)

TRIzol reagent (cat. no. DP424, Tiangen Biotech, Beijing, China) was utilized to extract total RNA of heart tissue. First-strand cDNA was transcribed using a PrimeScript^TM^ RT reagent kit with gDNA (cat. no. KR116-02, Tiangen Biotech). The qRT-PCR was conducted using SYBR green as the fluorescent dye (cat. no. FP205-02, Tiangen Biotech) in a reaction volume of 20 μL, consistent with the standard manufacturer instructions provided. The qRT-PCR analysis was performed in an ABI 7900Ht thermocycler (Applied Biosystems, Foster City, CA, USA). Relative gene expression levels were calculated using the 2^‐ΔΔCT^ method. Glyceraldehyde 3-phosphate dehydrogenase was quantified as the internal control. All primer sequences used in the present study, shown in Table 1, were synthesized by Shanghai Sangon Biotech Engineering (Shanghai, China).

**Table 1.** All Primer Sequences Used In Qrt-PCR Experiment In The Present Study

Gene	Primer	Sequence
IL-1β	Forward Primer	GCATCCAGCTTCAAATCTCGC
	Reverse Primer	TGTTCATCTCGGAGCCTGTAGTG
IL-6	Forward Primer	CCCCAATTTCCAATGCTCTCC
	Reverse Primer	CGCACTAGGTTTGCCGAGTA
TNF-Α	Forward Primer	CCCTCACACTCACAAACCACC
	Reverse Primer	CTTTGAGATCCATGCCGTTG
Collagen I	Forward Primer	GAGCGGAGAGTACTGGATCGA
	Reverse Primer	CTGACCTGTCTCCATGTTGCA
Collagen III	Forward Primer	TGCCATTGCTGGAGTTGGA
	Reverse Primer	GAAGACATGATCTCCTCAGTGTTGA
Fibronectin	Forward Primer	TGGAACTTCTACCAGTGCGAC
	Reverse Primer	TGTCTTCCCATCATCGTAACAC
GAPDH	Forward Primer	CCTCGTCCCGTAGACAAAATG
	Reverse Primer	TGAGGTCAATGAAGGGGTCGT

### Western blotting analyses

Total protein was obtained from heart tissues of mice by a protein extraction kit (cat. no. P0013B, Beyotime Biotechnology, Jiangsu, China). Protein concentrations were determined by a bicinchoninic acid protein assay kit (cat. no. P0012, Beyotime Biotechnology). A total of 50 μg of protein from each sample were separated by sodium dodecyl sulfate-polyacrylamide gel electrophoresis, then transferred to polyvinylidene difluoride membranes (EMD Millipore, Billerica, MA, USA). The membranes were then incubated with the appropriate primary antibody for more than 12 h at 4°C. Subsequently, the membranes were washed with Tris-buffered saline containing 0.1% Tween-20, then incubated with the appropriate secondary antibody (goat anti-rabbit IgG, cat no. B900210; 1:5000; or goat anti-mouse IgG, cat no. 15014; 1:5000; both from Proteintech Rosemont, IL, USA) for more than 1 h. An enhanced chemiluminescence detection kit and scanner were used to measure protein bands (Thermo Fisher Scientific, Waltham, MA, USA). The primary antibodies included: anti-IL-1β (cat. no. Ab200478; 1:1000; Abcam, Cambridge, MA, USA), anti-IL-6 (cat. no. ab6672; 1:1000; Abcam), anti-TNF-α (cat. no. ab1793; 1:1000; Abcam), anti-NF-κB (cat. no. CST-8242S; 1:1000; Cell Signaling Technology, Danvers, MA, USA), anti- phosphorylated (p)-NF-κB (cat. no. CST-3033S; 1:1000; Cell Signaling Technology), anti-collagen I (cat. no. ab34710; 1:1000; Abcam), anti-collagen III (cat. no. ab7778; 1:1000; Abcam), anti-Smad2/3 (cat. no. CST-5678S; 1:1000; Cell Signaling Technology), anti-p-Smad2/3 (cat. no. CST-9510S; 1:1000; Cell Signaling Technology), anti-Bcl-2 (cat. no. ab182858; 1:1000; Abcam), and anti-Bax (cat. no. ab32503; 1:1000; Abcam). Western blotting was performed at least 3 times in independent experiments. Image Lab 4.0.1 software was used to analyze the results.

### Statistical analyses

All data are presented as means ± standard deviations. Prism 7.0 software (GraphPad Software, San Diego, CA, USA) was utilized to conduct the statistical analyses. We used a one-way analysis of variance for comparing multiple groups. P values < 0.05 were considered statistically significant.

## Results

In the present study, we investigated the effect of LYC on cardiac dysfunction induced by ISO. The results indicated that treatment with LYC can ameliorate ISO-induced cardiac dysfunction. Furthermore, the LYC-mediated improvement of cardiac dysfunction induced by ISO may derive from the inhibition of inflammation, fibrosis, oxidative stress, and apoptosis in heart tissue, implicating LYC as a promising therapeutic compound in ISO-induced cardiac dysfunction.

### Administration of LYC alleviated cardiac dysfunction induced by ISO

**Figure 2.**
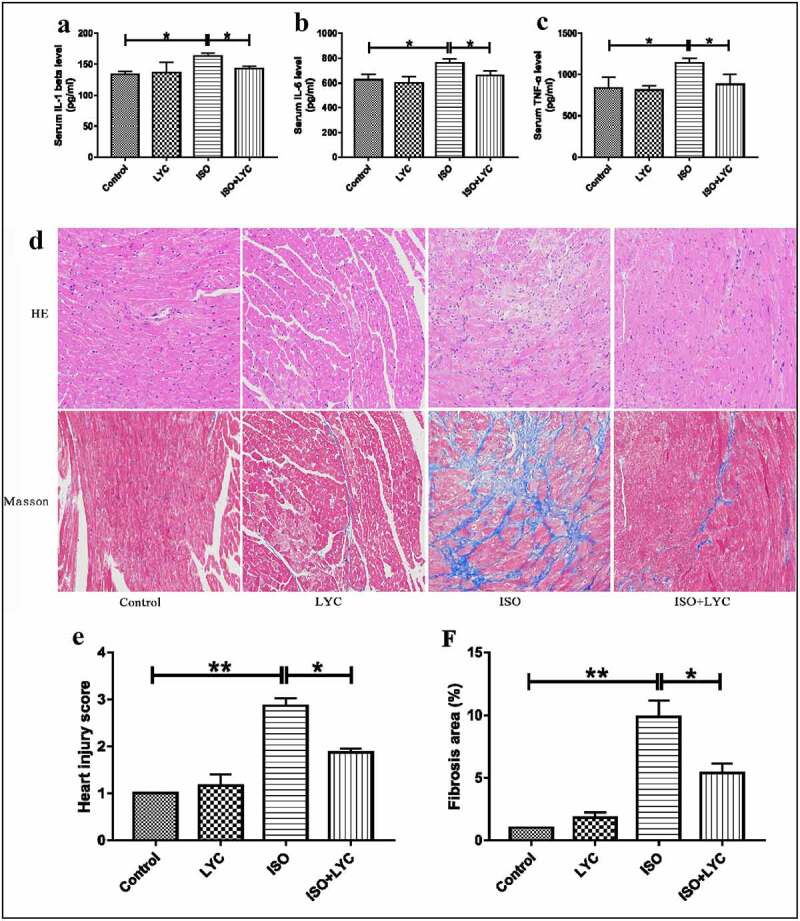
Treatment Of LYC Ameliorated Histopathological Injury In Heart Induced By ISO. (a,–c) The Levels Of Inflammatory Cytokines Like IL-1β, IL-6 And TNF-Α Were Assessed By Assay Of ELISA. (d) The Results Of H&E Staining And Massion Staining. (e) Heart Injury Score Was Used To Assess Degree Of Cardiac Injury. (f) Fibrosis Area (%) Was Used To Evaluate Degree Of Cardiac Fibrosis. *P < 0.05 And **P < 0.01 Mean Statistic Significance

A summary of treatments is displayed in Figure 1(a). Firstly, we performed echocardiography to assess the change of cardiac function induced by ISO in mice. The results show that cardiac function was markedly impaired by injection of ISO, while administration of LYC significantly inhibited this dysfunction, as evidenced by improvements of the ejection fraction and fractional shortening in the heart (Figure 1(b–d)). In addition, we detected the levels of specific cardiac injury biomarkers (CK-MB and LDH) in plasma to evaluate the degree of injury to the heart. Compared with the control group, the plasma levels of CK-MB and LDH in the ISO group increased significantly, while the increases were inhibited significantly by LYC (Figure 1(e,f)). We also measured the inflammatory cytokines, IL-1β, IL-6, and TNF-α, in plasma to assess any changes of inflammation. The results revealed that the injection of LYC significantly reduced the inflammation induced by ISO (Figure 2(a–c)). These data suggested that treatment with LYC alleviated the cardiac dysfunction induced by ISO.

### Treatment with LYC ameliorated histopathological injury in heart induced by ISO

Histopathological injury in the heart is a typical feature of ISO-induced cardiac injury. Using hematoxylin and eosin and Masson’s trichrome staining to appraise the pathological changes, we found that, compared with the control group, ISO obviously damaged cardiac structures, and contributed to edema and disordered myofilament arrangement in myocardial cells in mice, while the effects were significantly improved by injection of LYC (Figure 2(d,e)). Similarly, ISO increased the fibrotic area in the heart. When treated with LYC, the area of cardiac fibrosis was significantly reduced (Figure 2(d,f)). In the present study, the ‘heart injury score’ was assessed by edema, rupture, apoptosis, neutrophilic infiltration, contraction band necrosis, intramuscular bleeding, and ischemia. This score indicated that treatment with LYC ameliorated injury of heart tissue induced by ISO.

### Injection of LYC inhibited inflammation in heart tissue induced by ISO

**Figure 3.**
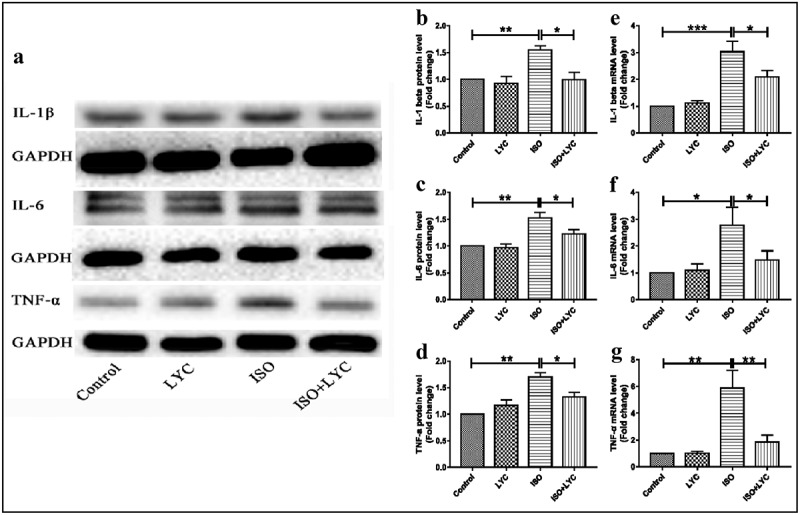
Treatment Of LYC Inhibited Inflammation In Heart Tissue Induced By ISO. (a) The Protein Bands Of IL-1β, IL-6 And TNF-Α Were Detected By Western Blotting. (b–d) Statistical Analyses Of Protein Levels Like IL-1β, IL-6 And TNF-Α. (e–g) Statistical Analyses Of Mrna Levels Of IL-1β, IL-6 And TNF-Α. *P < 0.05, **P < 0.01 And ***P < 0.001 Mean Statistic Significance

An abnormal inflammatory response in heart tissue contributes to ISO-induced cardiac injury. To elucidate the effect of treatment with LYC on the ISO-induced inflammatory response, the protein and mRNA levels of IL-1β, IL-6 and TNF-α in heart tissue of mice were evaluated by western blotting and qRT-PCR, respectively. Compared with the control group, the protein levels of these inflammatory cytokines were significantly upregulated in mice of the ISO group, while treatment with LYC significantly decreased these cytokines in ISO-treated mice (Figure 3(a–d)). Similar inhibitory effects of LYC on the mRNA levels of these inflammatory cytokines in heart tissue were also observed (Figure 3(e–g)).

**Figure 4.**
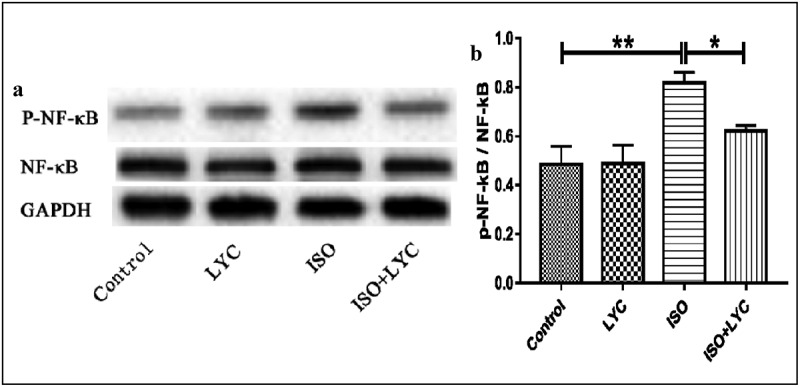
Treatment Of LYC Suppressed Cardiac Inflammation Partially By Inhibiting Activation Of The NF-Κb Signaling Pathway. (a,b) The Protein Level Of Activated NF-Κb Was Measured By Western Blotting. *P < 0.05 And **P < 0.01 Mean Statistic Significance

The NF-κB signaling pathway is well-known as a molecular mechanism to modulate the inflammatory response. Thus, we evaluated the effect of treatment with LYC on ISO-induced activation of the NF-κB signaling pathway in mouse hearts. Western blotting showed that treatment with LYC suppressed the phosphorylation of NF-κB induced by ISO, indicating that LYC treatment suppressed cardiac inflammation partially by inhibiting activation of the NF-κB signaling pathway (Figure 4(a,b)).

### Treatment with LYC reduced fibrosis in heart tissue induced by ISO

**Figure 5.**
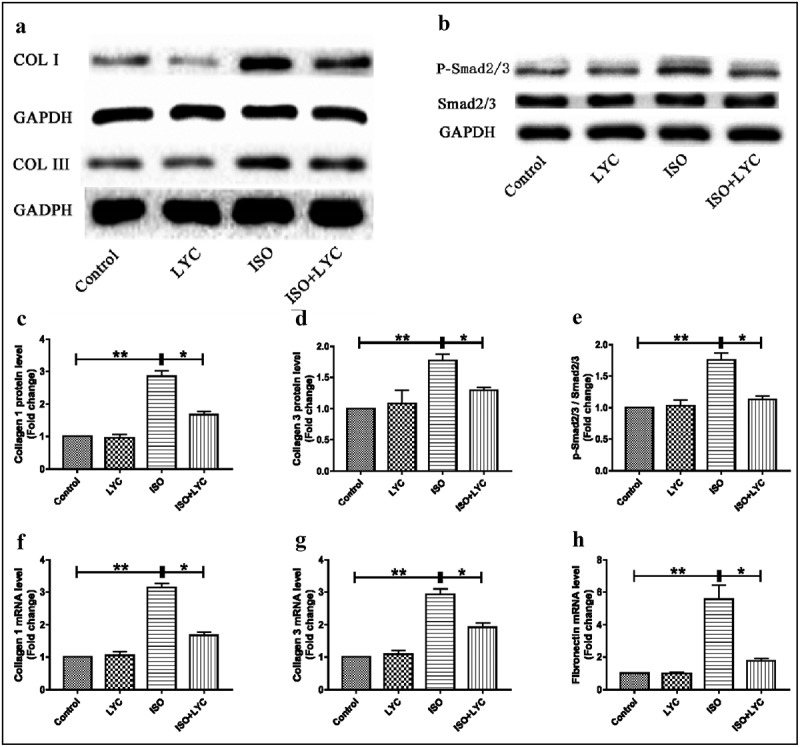
Treatment Of LYC Reduced Fibrosis In Heart Tissue Induced By ISO. (a) The Protein Bands Of Collagen I And Collagen III Were Detected By Western Blotting. (b) The Protein Bands Of Activated Smad2/3 Was Measured By Western Blotting. (c–e) Statistical Analyses Of Protein Levels Like Collagen I, Collagen III And P-Smad2/3. (f–h) Statistical Analyses Of Mrna Levels Of Collagen I, Collagen III And Fibronectin. *P < 0.05 And **P < 0.01 Mean Statistic Significance

Cardiac fibrosis is a major pathological change in ISO-induced cardiac dysfunction. Therefore, we investigated the effects of treatment with LYC on ISO-induced cardiac fibrosis. The expression levels of collagen I and collagen III increased significantly at both the mRNA and protein levels in heart tissue of mice challenged with ISO compared to those in the control group, while this effect was suppressed by treatment with LYC (Figure 5(a,c,d,f,g)). We also detected the fibronectin mRNA level and found that treatment with LYC significantly reduced the increased mRNA level induced by ISO (Figure 5(h)). The Smad signaling pathway was explored to elucidate the molecular mechanism in cardiac fibrosis. Western blotting revealed that treatment with LYC inhibited the phosphorylation of Smad2/3 induced by ISO, suggested that this treatment may have suppressed cardiac fibrosis through inhibiting activation of the Smad signaling pathway (Figure 5(b,e)).

### Administration of LYC inhibited oxidative stress in heart tissue induced by ISO

**Figure 6.**
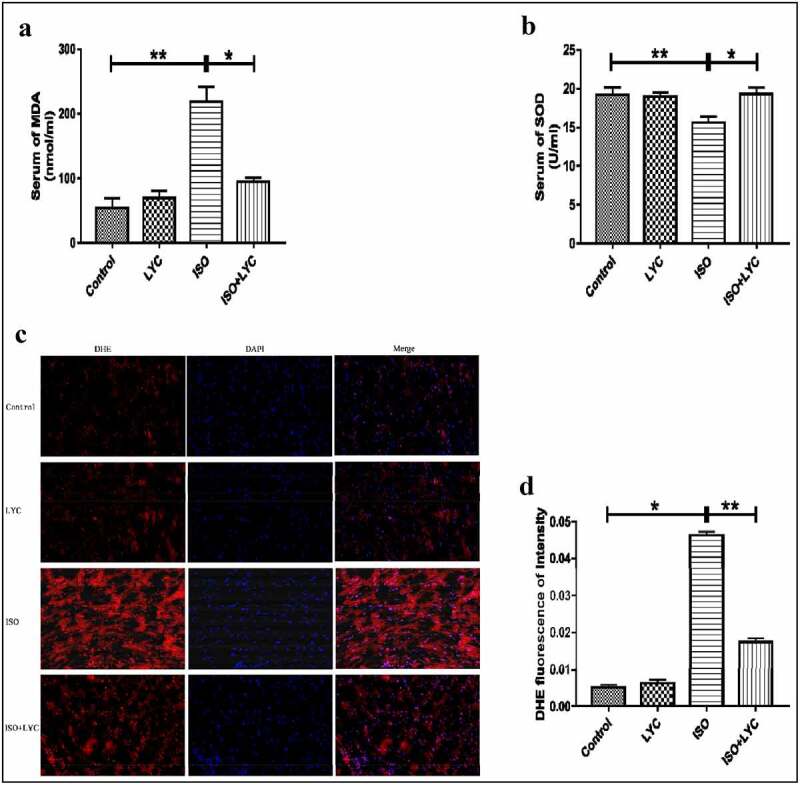
Treatment Of LYC Inhibited Oxidative Stress In Heart Induced By ISO. (a,b) The Concentration Of MDA And SOD Activity In Plasma Were Assessed By Specific Kits. (c) The Result Of Assay Of DHE Staining. D, The Level Of Total ROS Was Evaluated By DHE Fluorescence Of Intensity. *P < 0.05 And **P < 0.01 Mean Statistic Significance

Oxidative stress induced by ISO plays a vital role in the progression of cardiac dysfunction. To investigate whether treatment with LYC affected the level of oxidative stress induced by ISO, DHE staining was conducted. Compared with control mice, the total superoxide level measured by DHE staining was increased significantly in the ISO group, while it was suppressed in the ISO + LYC group (Figure 6(c,d)). We also measured the concentration of MDA and SOD activity in plasma to determine its redox status. Compared with the control group, the ISO group exhibited a higher level of MDA and lower SOD activity, while those changes were prevented by treatment with LYC (Figure 6(a,b)). In summary, treatment with LYC may protect against cardiac dysfunction induced by ISO through inhibiting oxidative stress.

### Injection of LYC suppressed apoptosis in heart tissue induced by ISO

**Figure 7.**
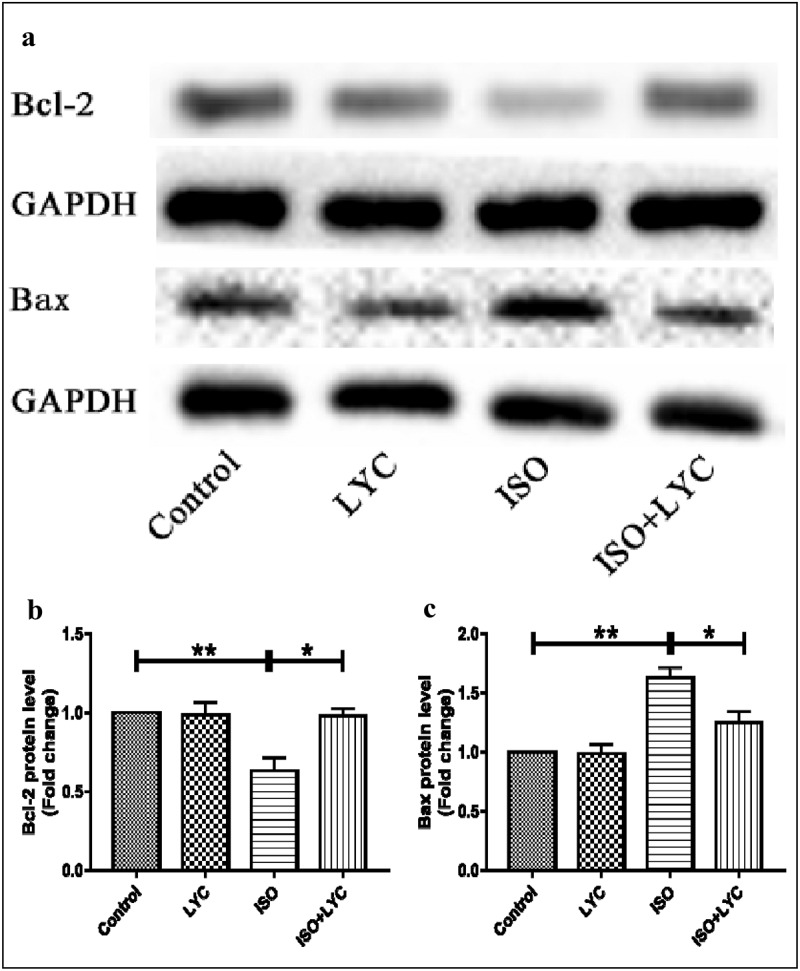
Treatment Of LYC Suppressed Apoptosis In Heart Induced By ISO. (a) The Protein Bands Of Bcl-2 And Bax Were Detected By Western Blotting. (b,c) Statistical Analyses Of Protein Levels Of Bcl-2 And Bax. *P < 0.05 And **P < 0.01 Mean Statistic Significance

Abnormal inflammatory responses often result in apoptosis of myocardial cells. Thus, we indirectly explored the effect of treatment with LYC on myocardial cell apoptosis stimulated by ISO. The anti-apoptotic marker Bcl-2, and pro-apoptotic marker Bax, were evaluated by western blotting. Compared with the control group, the protein level of Bcl-2 was decreased in the ISO group, but this effect was inhibited by treatment with LYC (Figure 7(a,b)). In contrast, the protein level of Bax increased significantly in the ISO group compared to the control group, while treatment of LYC suppressed this change (Figure 7(a,c)). These results suggested that treatment with LYC suppressed apoptosis in heart tissue induced by ISO.

## Discussion

As an alkaloid, LYC has been reported by several studies to have some significant anti-inflammatory, anti-fibrosis, antiviral, anti-aging, and anti-tumor activities [8–12]. LYC protects against acute lung injury induced by lipopolysaccharide mainly through inhibiting the production of pro-inflammatory mediators [25]. LYC also inhibits bleomycin-induced pulmonary fibrosis, principally via suppressing the inflammatory response, which displays a similar effect of danshensu in bleomycin-induced pulmonary fibrosis [26,30]. These studies suggested that LYC may serve as a new potential therapeutic compound to ameliorate inflammation and fibrosis in pulmonary diseases.

In addition to the above effects, LYC also exhibits antiviral activity. Recently, the novel coronavirus has been wreaking havoc around the world, contributing to higher morbidity and mortality of all countries. Thus, it is urgent to find novel therapeutic approaches for treating coronavirus disease 2019 (COVID-19). Jin et al. [24] found that LYC may serve as a potent non-nucleoside direct-acting antiviral against emerging coronavirus infections by suppressing the activity of viral RNA-dependent RNA polymerase. This suggests that LYC may be a candidate agent against the current COVID-19 pandemic. Similar antiviral effects of LYC were also discovered against flavivirus and Zika virus [22,23]. The anti-aging property of LYC derives from stabilizing the genome of human cells. This indicates that LYC may hold therapeutic potential for treating age-associated diseases [11].

Numerous studies have explored the anti-tumor activity of LYC, including against cancers such as colorectal, gastric, bladder, lung, breast, melanoma, multiple myeloma, osteosarcoma, and hematological malignancies. The molecular mechanism of the anti-tumor activity of LYC is mainly attributed to the selective induction of apoptosis in cancer cells [13–21].

In the present study, we evaluated the effect of LYC on ISO-induced cardiac dysfunction and found that it alleviated this dysfunction via suppressing inflammation, fibrosis, oxidative stress, and apoptosis. Furthermore, our results indicated that the NF-κB and Smad signaling pathways were involved in the cardioprotective effect of LYC. Inflammation, fibrosis, oxidative stress, and apoptosis have been reported to play important roles in the development of cardiac dysfunction induced by ISO [27]. Thus, we investigated the effect of LYC on these pathological changes. Echocardiography showed that treatment with LYC ameliorated cardiac dysfunction induced by ISO. The enzyme-linked immunosorbent assays revealed that LYC exerted an anti-inflammatory effect by reducing levels of the inflammatory cytokines, IL-1β, IL-6, and TNF-α. Because NF-κB activation can lead to transcription of these inflammatory cytokines [31], we assessed the effect of LYC and found that it inhibited the activation of NF-κB. Overall, these data suggested that the cardioprotective effect of LYC may be by inhibiting inflammation.

We also used Masson’s staining, western blotting, and qRT-PCR to show that treatment with LYC reduced cardiac fibrosis induced by ISO. Specifically, the fibrotic area in heart tissue was decreased and the expression levels of collagen I, collagen III, and fibronectin were down-regulated after treatment with LYC. The Smad signaling pathway has a well-known effect on the development of cardiac fibrosis [5]. Our findings showed that LYC inhibited the activation of Smad2/3, confirming the involvement of Smad signaling in its cardioprotective effect.

Furthermore, we explored the role of LYC in modulating ISO-induced oxidative stress. The results of DHE staining, and measuring the MDA level and SOD activity, revealed that treatment with LYC suppressed the increased oxidative stress induced by ISO. Finally, we investigated the effect of LYC on myocardial apoptosis stimulated by ISO. Changes in expression levels of the anti-apoptotic marker, Bcl-2, and pro-apoptotic marker, Bax, suggested that treatment with LYC inhibited myocardial apoptosis stimulated by ISO.

There are some limitations in the present study. Firstly, all experiments were performed in vivo. Thus, in vitro experiments need to be designed and conducted to comprehensively verify the results. Also, the molecular mechanisms involved in the present study were examined at a high level and need further in-depth investigation.

## Conclusion

The results of our study demonstrated that treatment with LYC protects against ISO-induced cardiac dysfunction by inhibiting inflammation, fibrosis, oxidative stress, and apoptosis. Moreover, our results indicated that the NF-κB and Smad signaling pathways are involved in the cardioprotective effects of LYC. Overall, the findings of our study suggest that LYC is a promising therapeutic compound to protect against cardiac dysfunction induced by ISO, and may, therefore, be a novel treatment for HF.
